# Prevalence of phubbing behaviour in school and university students in Spain

**DOI:** 10.3389/fpsyg.2024.1396863

**Published:** 2024-05-28

**Authors:** Noelia Barbed-Castrejón, Fermín Navaridas-Nalda, Oliver Mason, Javier Ortuño-Sierra

**Affiliations:** ^1^Educational Sciences Department, Universidad de La Rioja, Logroño, Spain; ^2^Psychology Department, Surrey University, Guildford, United Kingdom

**Keywords:** phubbing, adolescence, well-being, mental health, education

## Abstract

**Objective:**

This study examined the prevalence of phubbing behavior among school and university students in Spain and analyzed the correlation of phubbing with other indicators of psychological well-being and mental health.

**Methods:**

The study sample comprised a total of 1,351 school and university students, with ages ranging from 12 to 21 years. The study used the Phubbing Scale (PS), the Compulsive Internet Use Scale (CIUS), the Rosenberg Self-Esteem Scale (RSE), and the Interpersonal Emotion Regulation Questionnaire (IERQ) for data collection.

**Results:**

The results showed evidence of phubbing among approximately half of the students. Statistically significant differences were found based on gender and educational level in the Phone Obsession subscale and the PS total score, with male students and university students scoring higher in their respective parameters. In addition, phubbing was positively correlated with problematic internet use and negatively correlated with self-esteem.

**Conclusion:**

Phubbing behavior is highly prevalent among adolescents aged 12–21 years and is positively correlated with low self-esteem and problematic internet use. Developing strategies for addressing this widespread issue at early ages, particularly within the educational context, such as schools, is crucial for implementing preventive measures. The inappropriate use of technological devices, including smartphones, in schools has the potential to negatively affect students’ well-being and ability to adapt to school.

## Introduction

Adolescence is a critical developmental period with crucial consequences on individuals’ identity, self-concept, self-esteem, and values, among other aspects (Liu et al., 2022). Given these large-scale changes, adolescence is also a period of vulnerability to many psychological problems, including behavioral addictions (Rial Boubeta et al., 2015; Chotpitayasunondh and Douglas, 2016). Psychological problems prevailed among 10–20% of individuals aged between 12 and 16 years (de Vries et al., 2018), and three out of four mental health disorders among the adult population were first diagnosed during adolescence (Fusar-Poli, 2019). Adolescence is, thus, a critical period to prevent and address mental health issues (Irarrázaval et al., 2016; Moreno et al., 2019).

Adolescent smartphone owners are almost ubiquitous in Western Europe, with the use of smartphones including, but not limited to, work, study, and social interactions. In fact, Information and Communication Technologies (ICT), including the smartphone, have been integrated into classroom activities and educational programs (Cabero Almenara, 2015; Amores Valencia and De Casas Moreno, 2019; Navaridas-Nalda et al., 2020; Palacios-Rodríguez et al., 2023). Nonetheless, studies have questioned its adequacy in educational settings (Shahibi and Rusli, 2017; Twenge and Campbell, 2018; Burns, 2021). In this context, although some authors have recognized the benefits of incorporating technologies in the classroom, other authors’ contributions indicate contrasting views. These perspectives indicate the association between the use of smartphones and the specific behaviors that can adversely affect the teaching–learning process (Bisquerra Alzina and Chao Rebolledo, 2021). One such behavior is described using a relatively new term, phubbing; it is defined as the practice of ignoring the presence of others in a social environment while focusing on one’s smartphone (Karadaǧ et al., 2015; Chotpitayasunondh and Douglas, 2016). A phubber, a person who neglects others in favor of his smartphone, may lack the necessary self-control to appropriately use both phone and the internet (Arenz and Schnauber-Stockmann, 2023). This behavior can be characterized by a compulsive fear of missing out on other gratifying events and an inability to regulate the use of one’s phone (Chotpitayasunondh and Douglas, 2016); therefore, it may be described as an addiction-like behavior.

The use, or more precisely, the inappropriate use of smartphones, has been linked to symptoms of over-dependence, tolerance, and withdrawal (Fischer-Grote et al., 2019). Specifically, previous literature has shown adverse outcomes of phubbing, revealing that phubbing has a negative correlation with life satisfaction and a positive correlation with depression (Parmaksiz, 2021) as well as the feeling of loneliness and low self-esteem (Błachnio and Przepiorka, 2019). In addition, mental health issues, such as depression and anxiety, have been associated with the abuse of smartphones (Guazzini et al., 2019; Ergün et al., 2020). Furthermore, smartphone addiction has been shown to negatively moderate the relationship between self-regulation and well-being (Mascia et al., 2020). Other studies have revealed negative consequences of phubbing, including a decline in the quality of face-to-face relationships, a lower connection with individuals using smartphones in others’ presence (Przybylski and Weinstein, 2013; Zhang et al., 2023), and lowered empathy and limited social skills associated with phubbing behavior (Chotpitayasunondh and Douglas, 2016, 2018). Davey et al. (2018) indicated an alteration in eye contact and a reduction of social interactions due to phone misuse.

Studies have shown two notable negative consequences of phubbing behavior for romantic relationships (McDaniel and Coyne, 2016; Roberts and David, 2016; Al-Saggaf and MacCulloch, 2019; Ekimchik and Kryukova, 2022; Gorla et al., 2024) and family settings (Zhang et al., 2023). For instance, partner phubbing has been associated with an increase in the feeling of jealousy and depressive symptoms and a decrease in relationship satisfaction, although the reasons for these effects remain uncertain (McDaniel and Coyne, 2016; Roberts and David, 2016). In the context of adolescents, Zhang et al. (2023) found a positive correlation between teenage phubbing and conflicts with parents. Moreover, parental phubbing was related to depression in students of different educational levels in China (Kong et al., 2021; Hu et al., 2023; Mi et al., 2023).

The prevalence rates of phubbing generally appear to differ according to gender although this trend may depend on the culture and context. In general, most studies found higher rates of phubbing behavior among women (Anshari et al., 2016; Villafuerte-Garzón and Vera-Perea, 2018; Błachnio and Przepiorka, 2019; Balta et al., 2020; Escalera-Chávez et al., 2020; Błachnio et al., 2021). For example, the study of Anshari et al. (2016) on Brunei’s university students revealed that female students had higher prevalence rates than their counterparts. Another study conducted on college students in Mexico (Escalera-Chávez et al., 2020) indicated that women were more likely to exhibit phubbing behavior. Similarly, a study conducted on adolescents and adults in Turkey revealed higher prevalence rates for women than for men (Balta et al., 2020). Two studies conducted in South America showed results that deviated from these findings; the study conducted in Ecuador found a greater prevalence of phubbing among men (Villafuerte-Garzón and Vera-Perea, 2018), whereas research conducted in Peruvian universities indicated no gender-based differences (Ríos Ariza et al., 2021).

Many studies have investigated phubbing behavior in educational contexts, with results suggesting a high prevalence of phubbing among students. For instance, a prevalence rate of 45.2% was found in adolescents in Spain (Cebollero Salinas et al., 2022), and 88.8% of university students in Turkey reported engaging in phubbing behavior (Ahmed et al., 2023). Based on a phubbing scale, college students in India had a 49.3% prevalence of phubbing (Davey et al., 2018). Similar prevalence rates were found among medical college students in India (42.7%) (Purwar et al., 2023) and pharmacy college students in the United States (41.3%) (Lo et al., 2022). However, research on the effect of gender on phubbing manifestation did not yield consistent results. For example, a study conducted on Saint Petersburg university students in Russia concluded no phubbing behavior among the students (Dushkin and Barinova, 2023).

Considering the widespread prevalence of phubbing, the differences in this phenomenon according to demographics, and the various detrimental consequences of phubbing, this study examined the prevalence of phubbing among Spanish adolescent students. The specific objectives of the study were (a) to determine the prevalence of phubbing in adolescents, (b) to analyze the effect of gender and educational levels on phubbing manifestations, (c) to investigate the correlation between phubbing and other indicators of well-being and mental health issues, and (d) to analyze the incidence of phubbing in different educational levels.

## Methodology

### Participants and procedures

A total of 1,351 students aged 12–21 years participated in the study. Students were enrolled in different educational levels, including secondary education, high school, vocational training, and university degree programs. The participants were selected through convenience sampling, and surveys were conducted in their educational institutions. Individual participants completed the questionnaires within approximately 20 min. Among the participants, 81% responded using their smart devices (mobile phones or laptops), whereas the remaining completed on-paper questionnaires. The gender distribution of the participants was as follows: 54.25% were men, 42.78% were women, less than 1% answered “other,” and approximately 2% chose not to specify their gender. Among the participants, 20% were employed or engaged in internships, with studies not being their exclusive or primary activity. The present study was approved by the Ethical Committee of Research of University of La Rioja, Spain.

### Instruments

#### Phubbing scale (PS)

The PS developed by Karadağ is a questionnaire comprising 10 items, with each question rated on a five-point Likert scale ranging from 1 (indicating never) to 5 (indicating always). The PS quantifies the frequency and severity of the behavior of neglecting the people around while being preoccupied with an internet-connected device, specifically a smartphone (Karadaǧ et al., 2015). Blanca and Bendayan (2018) developed an adapted version of the PS for the Spanish population, analyzing its consistency with the original study, particularly regarding disruptions in communication and obsession with the smartphone, which are the two dimensions of the scale. They also found evidence that phubbing is associated with internet addiction (Blanca and Bendayan, 2018). The internal consistency of the scale was confirmed using McDonald’s omega coefficient, which was 0.787 for the total score and 0.705 and 0.709 for the subscales of Communication Disturbance and Phone Obsession, respectively.

#### Rosenberg self-esteem scale (RSE)

The RSE, developed by Rosenberg in 1965, is a widely used tool for measuring an individual’s self-esteem. The RSE consists of 10 statements about an individual’s self-concept and self-evaluation. Each statement is rated on a four-point scale ranging from “strongly agree” to “strongly disagree.” Generally, the higher the RSE score, the higher the individual’s perceived self-esteem (Ronseberg, 1965). This study used the Spanish version of the RSE (Martín-Albo et al., 2007), which had a McDonald’s omega coefficient of 0.798 for the total score.

#### Interpersonal emotion regulation questionnaire (IERQ)

The IERQ, developed by Hoffman, consists of 20 items divided into four factors: Enhancing Positive Affect, Perspective Taking, Soothing, and Social Modeling. These factors are related to an individual’s inclination to seek out others to amplify their feelings of happiness and joy, turn to others as a reminder to not worry and feel better comfort, and learn from others how to handle a specific situation (Hofmann et al., 2016), respectively. This study used the Spanish adaptation of the IERQ (D’Orey Roquete et al., 2023), which was confirmed to have internal consistency with a McDonald’s omega coefficient of 0.918 for the total score.

#### Compulsive internet use scale (CIUS)

The CIUS is a 14-item self-assessment scale designed to measure the severity of internet addiction and compulsive, pathological, or problematic internet use (PIU). The 14 items are rated on a four-point scale ranging from 0 (indicating never) to 4 (indicating very often). The present study used the CIUS adapted to the Spanish context (Lopez-Fernandez et al., 2019; Ortuño-Sierra et al., 2022), which had a McDonald’s omega coefficient of 0.912.

### Data analysis

The present study used descriptive statistics including the percentage distribution of the PS items. Distributions were reported according to gender and educational level. A multivariate analysis of variance (MANOVA) was conducted using educational level and gender as fixed variables and the PS items and total score as dependent variables. For the cases where MANOVA indicated statistical significant differences, an analysis of variance (ANOVA) was conducted to analyze specific differences within groups. Finally, the correlation between phubbing and other mental health indicators was analyzed.

## Results

### Descriptive statistics and percentage distribution of PS items

Table 1 shows the mean and standard deviation of the PS items according to gender (i.e., man, woman, and others), educational level (i.e., non-university and university), and the total sample of *N* = 1,351. In addition, the percentage of participants scoring 4 or 5, corresponding to the options “almost always” and “always,” were calculated, and the results are shown in Table 2. The results reveal that 74.5% of the participants indicated that always or almost always had their phones within their reach (item 6); among them, 79.7% were university students and 70.8% were non-university students. Regarding gender, 77% of men and 71.9% of women reported that they always or almost always had their phones within their reach (item 6). In addition, 42.6% of the participants revealed that they always or almost always checked their phones when they woke up in the morning (Item 7). By contrast, only 16.8% of participants always or almost always found their eyes wandering on their phones when they were with others (item 1), and 11.3% revealed that they had the perception of annoying others when they were busy with their phones (item 5). Furthermore, only 3.7% stated that they were always or almost always busy with their mobile phones when they were with their friends (item 2); 5.7% had a similar perception regarding their family (item 4).

**Table 1:** Descriptive statistics (means and standard deviations) of the Phubbing Scale items.

	Total Sample (*N* = 1,351)	Male students (*n* = 733)	Female students (*n* = 578)	Non-university students (*n* = 789)	University students (*n* = 562)
	*M* (*SD*)	*M* (*SD*)	*M* (*SD*)	*M* (*SD*)	*M* (*SD*)
Communication disturbance					
1. My eyes start wandering on my phone when I’m together with others	2.65 (0.91)	2.71 (0.92)	2.59 (0.89)	2.56 (0.92)	2.79 (0.89)
2. I am busy with my mobile phone when I’m with my friends	2.05 (0.78)	2.03 (0.78)	2.06 (0.79)	1.99 (0.78)	2.13 (0.77)
3. People complain about me dealing with my mobile phone	1.77 (0.95)	1.76 (0.94)	1.76 (0.96)	1.82 (0.98)	1.71 (0.89)
4. I’m busy with my mobile phone when I’m with my family	2.10 (0.85)	2.08 (0.86)	2.10 (0.82)	2.00 (0.87)	2.23 (0.81)
5. I think that I annoy my partner when I’m busy with my mobile phone (or family. if you do not have a partner)	2.00 (1.10)	1.95 (1.06)	2.05 (1.14)	2.07 (1.14)	1.89 (1.04)
Phone obsession					
6. My phone is within my reach	4.03 (1.02)	4.08 (0.98)	3.95 (1.05)	3.94 (1.11)	4.15 (0.86)
7. When I wake up in the morning. I first check the messages on my phone	3.07 (1.40)	3.32 (1.38)	2.79 (1.38)	2.66 (1.42)	3.66 (1.14)
8. I feel incomplete without my mobile phone	2.21 (1.12)	2.35 (1.17)	2.01 (1.02)	2.07 (1.10)	2.39 (1.11)
9. My mobile phone use increases day by day	2.04 (0.90)	2.10 (0.90)	1.97 (0.88)	1.92 (0.86)	2.22 (0.93)
10. The time allocated to social. Personal or professional activities decreases because of my mobile phone	1.95 (1.04)	1.92 (1.03)	1.97 (1.05)	1.91 (1.01)	2.01 (1.07)

**Table 2:** Number and percentage of participants who scored 4 or 5 for the Phubbing Scale items.

	Total sample (*N* = 1,351)	Male students (*n* = 733)	Female students (*n* = 578)	Non-university students (*n* = 789)	University students (*n* = 562)
	*n* (*%*)	*n* (*%*)	*n* (*%*)	*n* (*%*)	*n* (*%*)
Communication disturbance					
1. My eyes start wandering on my phone when I’m together with others	226 (16.8)	135 (18.4)	84 (14.7)	114 (14.6)	112 (19.9)
2. I am busy with my mobile phone when I’m with my friends	50 (3.7)	26 (3.5)	22 (3.8)	27 (4.4)	23 (4.1)
3. People complain about me dealing with my mobile phone	85 (6.3)	46 (6.3)	37 (6.4)	56 (7.1)	29 (5.2)
4. I’m busy with my mobile phone when I’m with my family	76 (5.7)	39 (5.3)	30 (5.2)	42 (5.4)	34 (6)
5. I think that I annoy my partner when I’m busy with my mobile phone (or family, if you do not have a partner)	153 (11.3)	71 (9.7)	74 (12.8)	103 (13.1)	50 (8.9)
Phone obsession					
6. My phone is within my reach	1,004 (74.5)	564 (77)	414 (71.9)	556 (70.8)	448 (79.7)
7. When I wake up in the morning, I first check the messages on my phone	575 (42.6)	366 (50.2)	194 (33.6)	240 (30.4)	336 (59.6)
8. I feel incomplete without my mobile phone	190 (14.1)	134 (18.3)	50 (8.7)	94 (11.9)	96 (17.1)
9. My mobile phone use increases day by day	80 (5.9)	49 (6.7)	27 (4.7)	34 (4.3)	46 (8.2)
10. The time allocated to social, personal or professional activities decreases because of my mobile phone	128 (9.5)	68 (9.3)	55 (9.5)	63 (8)	65 (11.6)

### Phubbing based on gender and educational level

To analyze the possible effect of gender and educational level on the manifestation of phubbing behavior, MANOVA was performed with gender and educational level as fixed factors and the PS total score and subscales of phubbing as dependent variables. The Wilks’ Lambda (λ) was used to detect statistically significant differences among the variables. The Partial Eta Square (*η*^2^) was used to analyze the effect size. The mean and standard deviations of the subscales and the total score based on gender, educational level, and the total sample are shown in Table 3. The *Wilks’* λ values indicated statistically significant differences in gender (*Wilks’* λ = 0.987, *p*-value <0.001, *η*^2^ = 0,013) and educational level (*Wilks’* λ = 0.931, *p*-value <0.001, *η*^2^ = 0,013).

**Table 3:** Descriptive statistics of the Phubbing Scale subscales and total score.

Phubbing scale subscales	Total sample (*N* = 1,351)	Male students (*n* = 733)	Female students (*n* = 578)		Non-university students (*n* = 789)	University students (*n* = 562)	
	*M* (*DT*)	*M* (*DT*)	*M* (*DT*)	*p*-value	*η^2^*	*M* (*DT*)	*M* (*DT*)	*p*-value	*η^2^*
Communication disturbance	10.56 (3.16)	10.54 (3.17)	10.55 (3.14)	0.871	0.000	10.44 (3.15)	10.75 (3.18)	0.081	0.002
Phone obsession	13.30 (3.79)	13.76 (3.73)	12.68 (3.68)	< 0.001	0.010	12.49 (3.76)	14.42 (3.40)	< 0.001	0.062
Total score	23.87 (5.95)	24.31 (5.98)	23.24 (5.86)	0.036	0.003	22.93 (5.94)	25.17 (5.72)	< 0.001	0.033

Subsequent ANOVA indicated statistically significant differences in the Phone Obsession subscale (*F* = 12.630, *p*-value <0.001, partial *η*^2^ = 0.010) and the PS total score (*F* = 4.416, p-value = 0.036, partial *η*^2^ = 0.003). In addition, ANOVA for educational level revealed statistically significant differences in the Phone Obsession subscale (*F* = 85.995, *p*-value <0.001, partial *η*^2^ = 0.062) and the PS total score (*F* = 44.341, *p*-value <0.001, partial *η*^2^ = 0.033). No statistical differences were found in the Communication Disturbance subscale for both gender and educational level.

### Correlation between phubbing and indicators of well-being, emotion regulation, and PIU

The correlation of the PS subscales and total score with different variables of well-being and emotion regulation was analyzed using Pearson’s correlation, and the results are presented in Table 4. The results indicate that all correlations were statistically significant, with the exception of the correlation between IERQ Enhancing Positive Affect and the PS Communication Disturbance subscale and that between IERQ Social Modeling and the RSE total score. The correlation coefficients of the PS and the other indicators ranged between 0.51 (PS total and CIUS total scores) and 0.045 (IERQ Enhancing Positive Affect and PS Communication Disturbance).

**Table 4:** Correlation between phubbing and other mental health indicators.

	1	2	3	4	5	6	7	8	9
PS total (1)	–								
PS communication disturbance (2)	0.836^**^	–							
PS phone obsession (3)	0.885^**^	0.485^**^	–						
IERQ enhancing positive affect (4)	0.090^**^	0.045	0.103^**^	–					
IERQ perspective taking (5)	0.076^**^	0.063^*^	0.066^*^	0.358^**^	–				
IERQ social modeling (6)	0.138^**^	0.080^**^	0.152^**^	0.559^**^	0.623^**^	–			
IERQ soothing (7)	0.123^**^	0.124^**^	0.091^**^	0.447^**^	0.650^**^	0.639^**^	–		
IERQ total (8)	0.132^**^	0.101^**^	0.125^**^	0.698^**^	0.820^**^	0.865^**^	0.861^**^	–	
CIUS total (9)	0.501^**^	0.408^**^	0.454^**^	0.063^*^	0.154^**^	0.180^**^	0.142^**^	0.170^**^	–
RSE total	−0.170^**^	−0.134^**^	−0.155^**^	0.097^**^	0.167^**^	0.037	0.060^*^	0.111^**^	−0.259^**^

^**^p-value < 0.01; ^*^p-value < 0.05.

## Discussion

Although phubbing behavior is a prevalent problem that affects adolescents’ well-being (Guzmán-Brand and Gelvez-García, 2022), evidence confirming its impact on adolescent populations remains limited. Therefore, the present study mainly aimed to analyze the prevalence of phubbing among Spanish adolescents and the possible effect of gender and educational level on the manifestation of phubbing behavior. In addition, the study analyzed the correlation between phubbing and other indicators of psychological well-being.

The results of the present study indicated that phubbing was prevalent among adolescents. For instance, three out of four participants reported that their phone was almost always within their reach; one in six reported, “My eyes start wandering on my phone when I’m together with others.” Indicators of both obsession and communication were prevalent. The overall results are consistent with those of recent studies (Davey et al., 2018; Cebollero Salinas et al., 2022; Ahmed et al., 2023; Purwar et al., 2023). Similar to the present study’s results, Davey et al. (2018) found a 49.3% prevalence of phubbing. Purwar et al. (2023) revealed that 42.7% in India exhibited phubbing behavior, while Lo et al. (2022) showed 41.3% prevalence of phubbing. However, some other studies have shown results that are incongruent with the abovementioned results. For example, Dushkin and Barinova (2023) did not find phubbing indicators among Russian students. The present study’s finding that phubbing is gaining prevalence among Spanish school and university students is of significant concern considering that previous literature has related phubbing with negative consequences such as a decline in face-to-face communications (Przybylski and Weinstein, 2013; Zhang et al., 2023).

Subsequently, the study analyzed the possible effects of gender and educational level on the manifestation of phubbing behavior. With regard to gender, previous studies indicated that women exhibit a higher prevalence of phubbing than men (Anshari et al., 2016; Villafuerte-Garzón and Vera-Perea, 2018; Błachnio and Przepiorka, 2019; Balta et al., 2020; Escalera-Chávez et al., 2020; Błachnio et al., 2021). The results of the present study did not agree with this finding considering the higher prevalence rate obtained for men.

In the present study, university students exhibited higher prevalence rates for Phone Obsession and the total score of Phubbing than those from lower educational levels. This result indicates that university students have a particularly high prevalence of phubbing, which agrees with another study that showed a prevalence rate of 88.8% among university students from Turkey (Ahmed et al., 2023). Alonso and Romero (2021) suggested that PIU increases with age among adolescents. Research has shown the negative consequences of phone addiction, specifically phubbing (Nikhita et al., 2015; Błachnio and Przepiorka, 2019), and the fact that addiction-related behaviors are likely to transcend to larger problems in adulthood (Anderson et al., 2017). Therefore, analyzing the development of phubbing and related phenomena during adolescence is crucial (Dahl and Bergmark, 2020) for implementing prevention strategies that can help individuals at an early stage. The use of technological devices in classrooms, including smartphones, which may lead to problematic behaviors such as phubbing, can hinder students’ ability to adapt to educational institutes. Therefore, detailed research on the possible adverse effects of smartphone usage in the teaching and learning processes is critical, as emphasized by other studies (Bisquerra Alzina and Chao Rebolledo, 2021). Such research will enable the implementation of preventive strategies in educational settings.

Finally, the present study analyzed the correlation between phubbing and different indicators of psychological well-being and mental health. Previous literature indicated that phubbing is negatively correlated with relevant aspects of well-being such as life satisfaction and self-esteem and positively correlated with mental health issues such as the feeling of loneliness, depression, and anxiety (Guazzini et al., 2019; Ergün et al., 2020). The results of the present study are consistent with these previous studies. The results indicated that phubbing was negatively correlated with adolescents’ self-esteem and positively correlated with PIU. Interestingly, all indicators of emotion regulation were either poorly correlated or positively correlated with phubbing. These findings imply that adolescents with higher levels of emotion regulation were at a higher risk of exhibiting phubbing behavior. This observation contradicts the idea that phubbing behavior causes a decrease in connection with others (Przybylski and Weinstein, 2013; Zhang et al., 2023) or lowers the levels of empathy and social skills (Chotpitayasunondh and Douglas, 2016, 2018) related to phubbing behavior. As indicated in previous studies, the use of ICT may allow and favor establishing social relationships and social networks (Arrivillaga et al., 2021; Pérez et al., 2021).

From these findings, it is evident that the results are contradictory and inconclusive. Therefore, further investigation is required to establish the causal relationships between phubbing and mental health issues and psychological well-being. Educational authorities are increasingly concerned about the widespread use of smartphones among students. Recent studies have highlighted the importance of comprehending how specific technologies could impact adolescent students (Pérez et al., 2021) and investigating the potential impact of technology use on adolescent mental health (Capilla Garrido et al., 2021; Lapierre and Zhao, 2024). Such insights can enable the development of strategies to protect individuals from the potential adverse effects of technology use (Cebollero-Salinas et al., 2022).

The present study has the following limitations. It relied on self-report instruments, which are based on certain assumptions and may include response bias. Thus, future research should introduce experimental data (e.g., behavioral or neuroimaging) and include other sources of information such as parents, teachers, or relatives. The study examined the effect of gender, rather than biological sex, on the manifestation of phubbing. Future studies could further explore this issue considering sex at birth or both as variables. In addition, due to the cross-sectional nature of the study, the cause–effect relationships could not be established. Finally, the study was conducted for a particular region in Spain; therefore, the results cannot be generalized to other regions.

Notwithstanding these limitations, this study provided insights into phubbing, a detrimental phenomenon related to the use of mobile phones, which are an integral part of the lives of adults and adolescents nowadays. Phubbing has been associated with potential mental health problems, which may have physical and psychological consequences, specifically during adolescence. In addition, phubbing may affect adolescents’ ability to adapt to school settings. Considering that studies analyzing phubbing behavior in the school context are still limited, the present study contributes valuable information about the prevalence of phubbing among Spanish adolescent students. Further research is crucial for a comprehensive understanding of the phenomenon of phubbing, particularly focusing on its potential impact on students’ socioemotional well-being and the ability to adjust to school.

## Data availability statement

The raw data supporting the conclusions of this article will be made available by the authors, without undue reservation.

## Ethics statement

The studies involving humans were approved by Research Ethics Committee. University of La Rioja. The studies were conducted in accordance with the local legislation and institutional requirements. Written informed consent for participation in this study was provided by the participants' legal guardians/next of kin. Written informed consent was obtained from the individual(s), and minor(s)’ legal guardian/next of kin, for the publication of any potentially identifiable images or data included in this article.

## Author contributions

NB-C: Investigation, Methodology, Visualization, Writing – original draft, Writing – review & editing. FN-N: Conceptualization, Methodology, Writing – review & editing. OM: Writing – original draft, Conceptualization. JO-S: Conceptualization, Methodology, Writing – review & editing.
